# Pathogen spectrum changes of respiratory tract infections in children in Chaoshan area under the influence of COVID-19

**DOI:** 10.1017/S0950268821001606

**Published:** 2021-07-30

**Authors:** Chuang-Xing Lin, Hao-bin Lian, Guang-Yu Lin, Dan-gui Zhang, Xiao-Ying Cai, Zhi-Wei Cai, Fei-qiu Wen

**Affiliations:** 1 Department of Pediatrics, The Second Affiliated Hospital of Shantou University Medical College, Shantou, Guangdong, China; 2 Translational Medicine Research Center, The Second Affiliated Hospital of Shantou University Medical College, Shantou, Guangdong, China; 3 Shenzhen Children's Hospital, Shenzhen, Guangdong, China

**Keywords:** Paediatrics, respiratory infections, virology (human) and epidemiology

## Abstract

From 24 January 2020 to 18 May 2020, Chaoshan took measures to limit the spread of coronavirus disease 2019 (COVID-19), such as restricting public gatherings, wearing masks and suspending classes. We explored the effects of these measures on the pathogen spectrum of paediatric respiratory tract infections in Chaoshan. Pharyngeal swab samples were collected from 4075 children hospitalised for respiratory tract infection before (May–December 2019) and after (January–August 2020) the COVID-19 outbreak. We used liquid chip technology to analyse 14 respiratory pathogens. The data were used to explore between-group differences, age-related differences and seasonal variations in respiratory pathogens. The number of cases in the outbreak group (1222) was 42.8% of that in the pre-outbreak group (2853). Virus-detection rates were similar in the outbreak (48.3%, 590/1222) and pre-outbreak groups (51.5%, 1468/2853; *χ*^2^ = 3.446, *P* = 0.065), while the bacteria-detection rate was significantly lower in the outbreak group (26.2%, 320/1222) than in the pre-outbreak group (44.1%, 1258/2853; *χ*^2^ = 115.621, *P* < 0.05). With increasing age, the proportions of respiratory syncytial virus (RSV) and cytomegalovirus (CMV) infections decreased, while those of *Mycoplasma pneumoniae* and adenovirus infections increased. *Streptococcus pneumoniae*, CMV and rhinovirus infections peaked in autumn and winter, while RSV infections peaked in summer and winter. We found that the proportion of virus-only detection decreased with age, while the proportion of bacteria-only detection increased with age (Table 2). Anti-COVID-19 measures significantly reduced the number of paediatric hospitalisations for respiratory tract infections, significantly altered the pathogen spectrum of such infections and decreased the overall detection rates of 14 common respiratory pathogens. The proportion of bacterial, but not viral, infections decreased.

## Background

As one of the most common paediatric diseases, respiratory tract infection is a major public health problem in both developing and developed countries and poses a massive economic burden globally [1–4]. The pathogen spectrum of epidemic respiratory tract infections differs among different geographic locations according to the climatic environment [5–8]. The Chaoshan area, located in Southeastern China, includes four cities, namely, Shantou, Shanwei, Chaozhou and Jieyang and has a large population. This area has a typical subtropical monsoon climate with hot and humid summers and cold and humid winters. Therefore, the pathogen spectrum of respiratory infections in children in this area has its own unique characteristics. The spread and prevalence of respiratory pathogens in 2020 has been considerably different from that in previous years owing to the prevention and control measures taken against coronavirus infectious disease 2019 (COVID-19), such as the suspension of classes, restrictions on public gatherings and wearing of masks. The purpose of this study was to explore the effects of the anti-COVID-19 measures on the pathogen spectrum of respiratory tract infections in children in the Chaoshan area.

## Materials and methods

### Study subjects and ethics statement

In the present study, pharyngeal swabs were collected from 4075 children (ages, 1 month to 14 years, mean age: 2 years and 6 months, median age: 1 year) who were treated for respiratory tract infections in Children's Hospital of the Second Affiliated Hospital of Shantou University Medical College, between May 2019 and August 2020. This hospital is the largest children's medical centre in Chaoshan area. The inclusion criteria were paediatric in-patients with clinically diagnosed respiratory tract infection. The diagnostic criteria for respiratory tract infection consisted of cough, expectoration and any one of the following three conditions: (1) fever, (2) increase in the total leucocyte count and/or proportion of neutrophils and (3) wet rales appearing in the lungs and/or inflammatory infiltrative lesions in the lungs on X-ray examination. The exclusion criteria were as follows: (1) newborns less than 28 days old, (2) inadequate pharyngeal swab samples, i.e. too few sampled cells or insufficient nucleic acid extraction, (3) incomplete clinical data, for example, lack of medical records or clinical diagnosis, (4) chest X-ray changes in respiratory tract caused by non-infectious causes such as pulmonary embolism, heart failure, pulmonary oedema and lung cancer. With 1 January 2020 as the boundary, the children included in the study were divided into two groups: the pre-outbreak group (May 2019 to December 2019) and the outbreak group (January 2020 to August 2020).

All the specimens were collected with the written consent of the parents, and the study protocol was approved by the ethics committee of the Second Affiliated Hospital of Shantou University Medical College.

### Specimen collection

Upon admission, patients aged greater than 2 years were instructed to gargle with warm saline twice and then, disposable aseptic pharyngeal swabs were used to gently and rapidly wipe the secretions on the palatine arch, pharynx and tonsils on both the right and left sides. After sampling, the pharyngeal swabs were quickly placed in collection tubes, which were immediately sealed using the tube covers, marked with the sampling date and patient name and number, stored in a refrigerator at 2–8 °C and analysed within 24 h.

### Nucleic acid-based pathogen detection

The respiratory swabs were sent to Guangzhou Da'an Clinical Examination Center, where they were subjected to total viral nucleic acid extraction via the magnetic bead method by using the KingFisher automated nucleic acid extraction instrument and the viral total nucleic acid extraction kit from Guangzhou Meiji Biotechnology Co. Ltd. [9], according to the manufacturers' instructions. After the extraction, nucleic acids of respiratory pathogens were detected using multiplex polymerase chain reaction (PCR) [10] and Luminex suspension liquid chip technology [11]. This technology integrates flow cytometry, encoding microspheres, lasers and digital signal processing, enabling the rapid, sensitive and specific detection of different pathogens. The diagnostic sensitivity and specificity of liquid chip technology are similar to those of PCR. Simultaneous detection of the following 14 main respiratory pathogens was performed: influenza A virus (FluA), influenza B virus (FluB), parainfluenza virus (PIV), human rhinovirus (HRV), adenovirus (ADV), respiratory syncytial virus (RSV), human metapneumovirus (hMPV), bocavirus (BoV), cytomegalovirus (CMV), *Haemophilus influenzae* (HI), *Streptococcus pneumoniae* (SP), *Moraxella catarrhalis* (MC), *Mycoplasma pneumoniae* (MP) and *Bordetella pertussis* (BP).

### Statistical methods

Data were statistically analysed using SPSS *v*23.0. Count data were expressed as cases and percentages. The chi-square test was used to compare the rate of pathogen detection in each group. Double-tailed probability was used in all tests, and a *P* value below 0.05 was considered statistically significant.

## Results

### General information

Of the 4075 cases in the present study, 2853 cases were included in the pre-outbreak group, and 1222 cases were included in the outbreak group (Table 1). The number of cases in the outbreak group was lower than that in the pre-outbreak group (at 57.2%). The positive rate of pathogen detection was significantly higher in the pre-outbreak group (74.5%, 2125/2853) than in the outbreak group (61.0%, 746/1222; *χ*^2^ = 74.191, *P* < 0.05). The virus detection rate did not significantly differ between the outbreak (48.3%, 590/1222) and pre-outbreak groups (51.5%, 1468/2853; *χ*^2^ = 3.446, *P* = 0.065). In contrast, the bacterial detection rate was significantly lower in the outbreak group (26.2%, 320/1222) than in the pre-outbreak group (44.1%, 1258/2853; *χ*^2^ = 115.621, *P* < 0.05).

**Table 1.** Comparison of infectious pathogens between the outbreak and pre-outbreak groups

Pathogen detected	Outbreak group (%)	Pre-outbreak group (%)	*χ*^2^	*P*
None	476 (39.0)	728 (25.5)	74.191	<0.05
Viral only	426 (34.9)	867 (30.4)	7.898	<0.05
Bacterial only	156 (12.8)	657 (23.0)	56.419	<0.05
Bacterial and viral	164 (13.4)	601 (21.1)	32.791	<0.05
Total	1222 (100)	2853 (100)		

### Respiratory pathogen detection

**Fig. 1.**
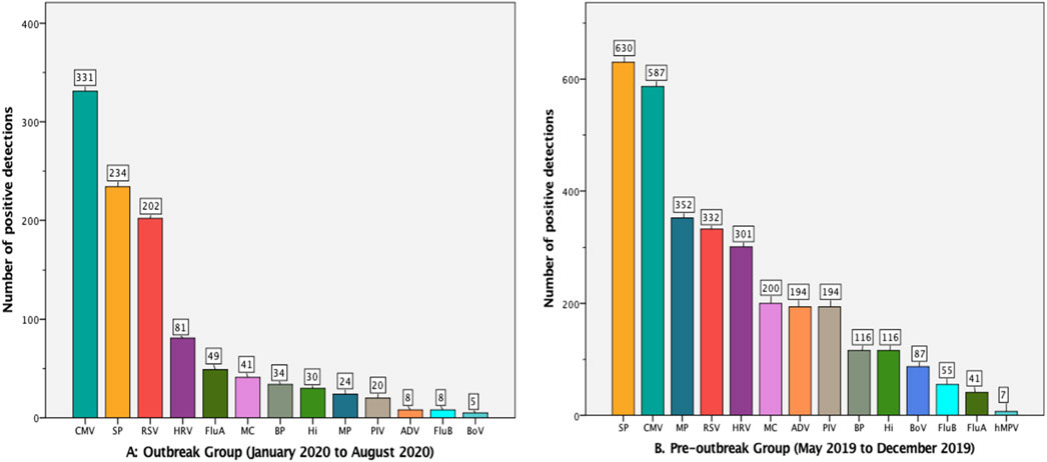
Distribution of pathogens detected in the outbreak and pre-outbreak groups.

The top five pathogens detected in the outbreak group were CMV (27.1%, 331/1222), SP (19.1%, 234/1222), RSV (16.5%, 202/1222), HRV (6.6%,81/1222), FluA (4.0%,49/1222; Fig. 1a), while the top five pathogens in the pre-outbreak group were SP (22.1%, 630/2853), CMV (20.6%, 587/2853), MP (12.3%, 352/2853),RSV (11.6%,332/2853) and HRV (10.6%,301/2853; Fig. 1b). The positive rates of detection of CMV, SP, RSV, MP, HRV and FluA significantly differed between the outbreak and pre-outbreak groups (*χ*^2^ = 20.787, 4.405,17.991, 109.929, 15.489 and 26.219 respectively; *P* < 0.05 for all). The other pathogens also statistically differed between the two groups (*P* < 0.05 for all), except hMPV (*P >* 0.05). Only seven cases of hMPV were detected in total; no case of hMPV was detected in the outbreak group.

### Respiratory pathogens and age

**Fig. 2.**
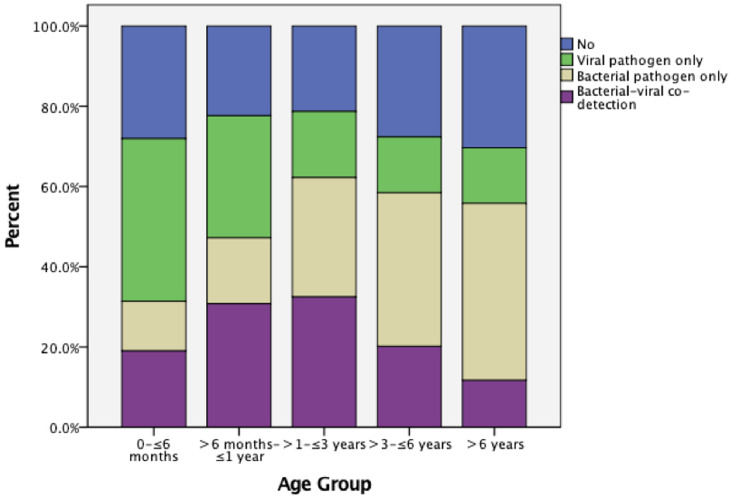
Pathogen detection results among children with respiratory tract infections stratified by age.

To study the changes in the respiratory pathogen spectrum with age, we divided all the study subjects into the following age groups: 0 to ≤6 months, >6 months to ≤1 year, >1 year to ≤3 years, >3 years to ≤6 years and >6 years. We assessed the proportions of the detection of a viral pathogen only, a bacterial pathogen only, both bacterial and viral pathogens and no pathogen at all in different age groups (Fig. 2). We found that the proportion of virus-only detection decreased with age, while the proportion of bacteria-only detection increased with age (Table 2).

**Table 2.** Pathogen detection results among children with respiratory tract infections stratified by age

Pathogen	Age group					*χ*^2^	*P*
	0 to ≤6 months (%)	>6 months to ≤1 year (%)	>1 to ≤3 years (%)	>3 to ≤6 years (%)	>6 years (%)		
No	306 (30.5)	287 (26.1)	262 (26.7)	194 (34.0)	155 (36.8)	26.643	<0.005
Viral pathogen only	475 (47.3)	419 (38.1)	218 (22.2)	105 (18.4)	76 (18.1)	257.172	<0.005
Bacterial pathogen only	91 (9.1)	142 (12.9)	247 (25.2)	181 (31.8)	152 (36.1)	243.821	<0.005
Bacterial−viral co- detection	132 (13.1)	251 (22.8)	254 (25.9)	90 (15.8)	38 (9.0)	94.912	<0.005
Total	1004 (100.0)	1099 (100.0)	981 (100.0)	570 (100.0)	421 (100.0)		

**Fig. 3.**
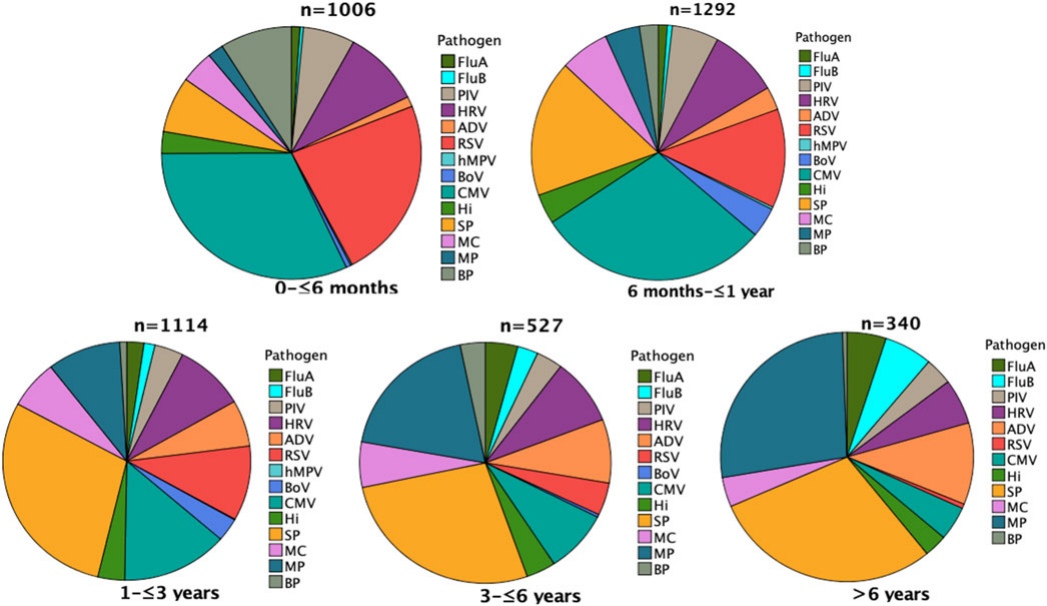
Distribution of detected pathogens among different age groups.

We also assessed the proportion of the detection of individual pathogens in each age group (Fig. 3 and Table 3). With increase in age, the detection rates of RSV and CMV gradually decreased (*χ*^2^ = 196.668, 330.742, respectively; *P* < 0.005 for all), while those of MP and ADV increased (*χ*^2^ = 214.657, 65.691, respectively; *P* < 0.005 for all).

**Table 3.** Distribution of detected pathogens among different age groups

Pathogen	Age group					*χ*^2^	*P*
	0 to ≤6 months (%) (*N* = 1004)	>6 months to ≤1 year (%) (*N* = 1099)	>1 to ≤3 years (%) (*N* = 981)	>3 to ≤6 years (%) (*N* = 570)	>6 years (%) (*N* = 421)		
FluA	11 (1.1)	15 (1.4)	25 (2.5)	22 (3.9)	17 (4.0)	23.623	<0.005
FluB	4 (0.4)	8 (0.7)	16 (1.6)	14 (2.5)	21 (5.0)	49.438	<0.005
PIV	65 (6.5)	77 (7.0)	42 (4.3)	18 (3.2)	12 (2.9)	21.573	<0.005
HRV	98 (9.8)	114 (10.4)	103 (10.5)	47 (8.2)	20 (4.8)	14.378	>0.005
ADV	13 (1.3)	38 (3.5)	70 (7.1)	45 (7.9)	36 (8.6)	65.691	<0.005
RSV*	234 (23.3)	162 (14.7)	113 (11.5)	23 (4.0)	2 (0.5)	196.668	<0.005
hMPV	2 (0.2)	4 (0.4)	1 (0.1)	0 (0.0)	0 (0.0)	4.395	>0.005
BoV	6 (0.6)	49 (4.5)	35 (3.6)	2 (0.4)	0 (0.0)	63.410	<0.005
CMV*	321 (32.0)	383 (34.8)	155 (15.8)	44 (7.7)	15 (3.6)	330.742	<0.005
HI	28 (2.8)	49 (4.5)	39 (4.0)	20 (3.5)	10 (2.4)	6.496	>0.005
SP*	72 (7.2)	224 (20.4)	325 (33.1)	143 (25.1)	100 (23.8)	209.070	<0.005
MC	42 (4.2)	81 (7.4)	73 (7.4)	32 (5.6)	13 (3.1)	19.842	<0.005
MP*	20 (2.0)	57 (5.2)	107 (10.9)	100 (17.5)	92 (21.9)	214.657	<0.005
BP*	90 (9.0)	31 (2.8)	10 (1.0)	17 (3.0)	2 (0.5)	113.924	<0.005
Total	1006 (100.2)	1292 (117.6)	1114 (113.6)	527 (92.5)	340 (80.8)		

Pathogens labelled (*) showed significant differences among different age groups.

### Seasonal variations in respiratory pathogens

**Fig. 4.**
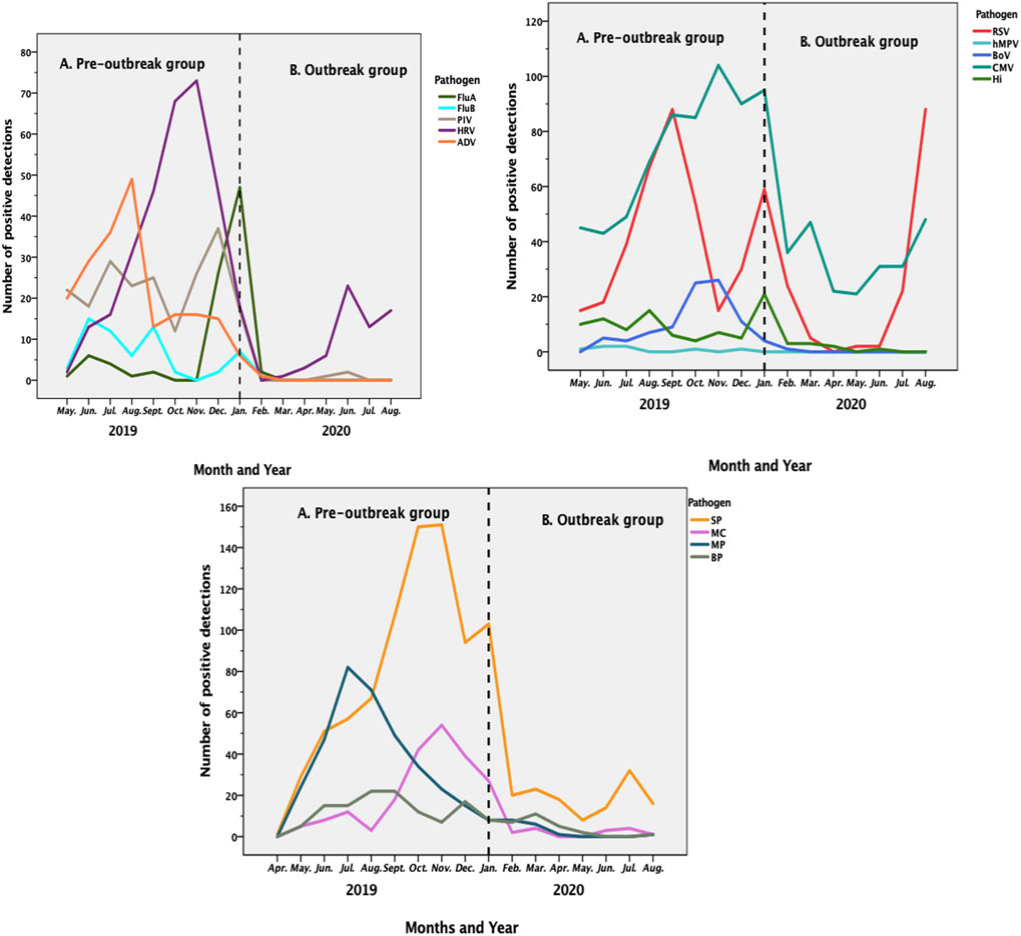
Seasonal variations in the detection of respiratory pathogens.

In this study, the four seasons are defined as follows: spring is March, April and May, summer is June, July and August, autumn is September, October and November and winter is December, January and February. We found that SP, CMV and HRV infections peaked in autumn and winter in 2019 and decreased after the COVID-19 outbreak (Fig. 4). RSV infections peaked in summer and winter in 2019 and summer in 2020, and the proportion of RSV infections decreased in the spring of 2020, i.e. after the outbreak. MP and ADV infections peaked in the summer of 2019 and then gradually decreased.

## Discussion

The present study showed that the prevention and control measures taken against COVID-19 significantly reduced the number of children hospitalised because of respiratory tract infections, changed the pathogen spectrum of respiratory tract infections and decreased the proportion of bacterial infections, but did not significantly change the proportion of viral infections.

Acute respiratory infection is an important cause of morbidity and mortality in children under 5 years of age [12, 13]. Furthermore, over a period of more than 50 years, there has been no significant change in the mortality rate of acute respiratory infections [4, 14, 15]. Therefore, in developing countries, including China, acute respiratory infection remains an important cause of death in children [16]. From 24 January 2020 to 18 May 2020, the Chaoshan area took measures to limit the spread of COVID-19, such as restricting public gatherings, wearing masks in public place and suspending classes. This paper studied the impact of these measures on the pathogen spectrum of respiratory tract infections in children in the Chaoshan area. A total of 14 respiratory pathogens were investigated during a period of 16 months. We found that the number of cases of respiratory tract infections during the 8 months after the COVID-19 outbreak (January to August 2020) was only 57.2% of the total number of cases in the 8 months before the outbreak (May−December 2019). Hence, we believe that the prevention and control measures taken during the COVID-19 pandemic significantly reduced the number of children who needed to be hospitalised due to respiratory tract infections.

In the outbreak group, respiratory pathogens were detected in 61.0% of the subjects. This rate is similar to the respiratory pathogen-detection rate (60.3%) among children in Dongguan City, another subtropical area [17], but it is lower than the rate in the pre-outbreak group (74.5%). These results imply that the respiratory pathogen spectrum of children in the Chaoshan area changed during the COVID-19 outbreak, and the proportion of respiratory infections caused by 14 common pathogens decreased. Notably, the proportion of viral pathogens did not change significantly, with virus detection rates of 48.3% in the outbreak group and 51.5% in the pre-outbreak group. Moreover, the virus detection rate in our study is similar to that found in our hospital in previous years (54.9%) [4].

The top five pathogens detected in the outbreak group were CMV (27.1%), SP (19.1%), RSV (16.5%), HRV (6.6%) and FluA (4.0%). The top five pathogens detected in the pre-outbreak group were SP (22.1%), CMV (20.6%), MP (12.3%), RSV (11.6%) and HRV (10.6%). The rate detection of most pathogens decreased in the outbreak group, compared with the pre-outbreak group. However, the rate detection of CMV and RSV increased in the outbreak group, compared with the pre-outbreak group. The rate detection of RSV increased from 11.6% (332/2853) in the pre-outbreak group to 16.5% (202/1222) in the outbreak group. Nevertheless, the anti-COVID-19 measures did appear to reduce the spread of RSV infections, as the number of actual cases of RSV infection reduced from 332 in the pre-outbreak group to 202 in the outbreak group, amounting to a decrease of 39.2%. RSV is mainly transmitted by respiratory droplets [18]. The virus particles are small, and ordinary masks cannot completely block its transmission, so the prevention and control measures may have had less of an impact on RSV infections than on other pathogens, such as bacteria. CMV is an opportunistic pathogen that is transmitted through a variety of routes, including sexual transmission, blood contact and inhalation of or contact with body fluids [19], so the transmission of CMV is also less affected by epidemic prevention and control measures. SP is a commensal bacterium in the human respiratory tract, with a high carrying rate among the general population [20–22]. It can be transmitted by droplets and aerosols [20], so limiting contact with people and wearing masks can significantly reduce its incidence. Another important reason is that there is no routine vaccination for SP in the Chaoshan area.

This study showed that with increase in age, the proportions of RSV and CMV infections gradually decreased, while the proportions of MP, SP and ADV infections increased. Consistent with our findings, a study in the United States found that RSV is the pathogen with the highest detection rate among children under 5 years old with pneumonia; the detection rate of RSV was as high as 37%, and it gradually decreased with age [14]. Some authors believe that the gradual decrease in the infection rates of RSV and CMV with age may be related to the low autoimmunity of very young children [9]. The proportions of MP infection gradually increased with age, which is consistent with a study from Dongguan [17] and may be related to the development of the normal respiratory microflora. In this study, the proportion of ADV infections increased with age. However, other studies have shown that the proportion of ADV infections does not increase with age, and there is no inevitable relationship between the two [4, 14, 23]. Our result may be related to the small number of ADV infection cases detected in this study and the great difference in the number of children in the different age groups.

SP, CMV and HRV infections peaked in autumn and winter in 2019, and the proportions of these infections decreased after the pandemic prevention and control measures were put in place. RSV infections peaked in summer and winter in 2019 and summer in 2020 and decreased in spring in 2020, probably due to the prevention and control measures. MP and ADV infections peaked in the summer of 2019 and then decreased gradually. Of course, infections by various pathogens are also related to the climate of Chaoshan area, which is a typical subtropical monsoon climate with hot and humid summers and cold and humid winters. This environment with high humidity may be beneficial to the transmission, colonisation and invasion of RSV, ADV and MP [17].

This study has some limitations. First, the study lasted for only 16 months, which is a relatively short time. A total of 4075 cases were included in this study, with 1 January 2020 as the boundary to divide the cases into two groups: the pre-outbreak group with 2853 cases and the outbreak group with 1222 cases. Thus, the data for each group were collected over a period of 8 months. Because the study period was less than 2 years, our data cannot be compared with data from the same region collected at the same time in different years, but can only be compared with data from the same region during the same length of time. Therefore, extending the research time may lead to more meaningful results. Second, this study only tested for the 14 most common respiratory pathogens in this region and did not analyse other pathogens.

## Conclusion

This study shows that the prevention and control measures taken against the COVID-19 pandemic significantly reduced the number of children who required to be hospitalised because of respiratory tract infections, significantly altered the pathogen spectrum of respiratory tract infections and decreased the positive rate of detection of respiratory tract pathogens. Among the 14 most common respiratory pathogens, the proportion of bacterial infections decreased, but the proportion of viral infections did not decrease significantly.

## Data Availability

The data that support the findings of this study are available from the corresponding author, Fei-qiu Wen, upon reasonable request.
